# Reindeer and the quest for Scottish enlichenment

**DOI:** 10.1177/20416695231218520

**Published:** 2023-12-15

**Authors:** Nathaniel J. Dominy, Catherine Hobaiter, Julie M. Harris

**Affiliations:** Departments of Anthropology and Biological Sciences, 3728 Dartmouth College, Hanover, NH, USA; School of Psychology and Neuroscience, 7486 University of St Andrews, St Andrews, Fife, UK; School of Psychology and Neuroscience, 7486 University of St Andrews, St Andrews, Fife, UK

**Keywords:** color, light, sensory plasticity/adaptation, visual search

## Abstract

In the hall of animal oddities, the reindeer (*Rangifer tarandus*) is the only mammal with a color-shifting tapetum lucidum and the only ruminant with a lichen-dominated diet. These puzzling traits coexist with yet another enigma––ocular media that transmit up to 60% of ultraviolet (UV) light, enough to excite the cones responsible for color vision. It is unclear why any day-active circum-Arctic mammal would benefit from UV visual sensitivity, but it could improve detection of UV-absorbing lichens against a background of UV-reflecting snows, especially during the extended twilight hours of winter. To explore this idea and advance our understanding of reindeer visual ecology, we recorded the reflectance spectra of several ground-growing (terricolous), shrubby (fruticose) lichens in the diets of reindeer living in Cairngorms National Park, Scotland.

The Scottish Highlands host an unrivaled diversity of lichens, but finding *Cladonia rangiferina* in March isn’t easy. Heather-bashing on Scotland's “hills” is arduous enough, but it is even more difficult when the prize is a carpet of whitish vegetation that resembles snow from a distance. Lichens may not seem the most desirable food, but spongy beds of bushy off-white *C. rangiferina*, or reindeer “moss,” are integral to the diet and survival of reindeer in Cairngorms National Park and elsewhere, sustaining them during the winter months. To our eyes, *C. rangiferina* blends in amongst the snow and granite outcroppings, but that may not be the case for the reindeer that exploit them.

Reindeer are the only mammals with a color-shifting tapetum lucidum, the retinal tissue responsible for “eye shine.” This mirror-like tissue reflects light entering the retina, giving photoreceptors a second opportunity for photon capture (Vee et al., 2022). It is a common trait among nocturnal animals because it enhances visual sensitivity under dim light, but the color of reflected light is normally constant. For reindeer, however, it changes seasonally, from a typical mammalian golden hue during the summer months to a vivid blue during the winter months (Figure 1a), only to reverse itself again with the onset of summer (Fosbury & Jeffery, 2022; Stokkan et al., 2013). The significance of this plasticity is uncertain, but the reflectivity of the winter tapetum extends into the ultraviolet (UV) range (Figure 1b), which seems related to another enigma of reindeer eyes––their cornea and lens. Typically, these tissues block UV wavelengths, protecting the eyes of day-active mammals. But those of reindeer transmit up to 60% of UV light, which is enough to excite the cones responsible for color vision (Hogg et al., 2011). To see UV light is puzzling enough, but what are the benefits of enhancing this ability during the circum-Arctic winter?

**Figure 1.**
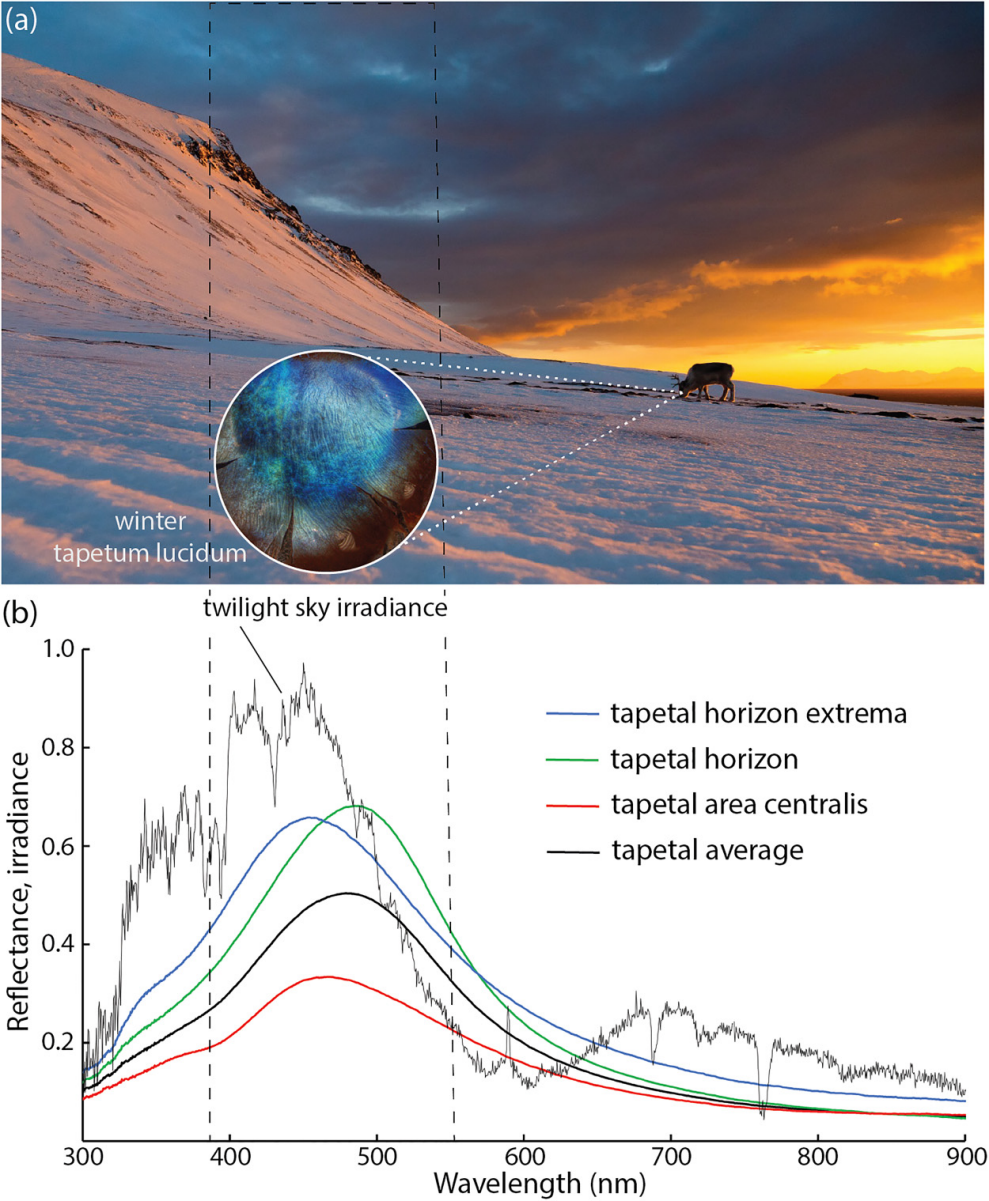
(a) A reindeer forages on ground-dwelling (terricolous) lichens at the onset of twilight in Svalbard, Norway (photograph by Espen Bergersen, reproduced with permission). (b) Twilight irradiance spectrum and tapetal reflectance spectra (winter phenotype; data source: Fosbury & Jeffery, 2022).

As any winter-sports enthusiast knows, increased exposure to UV from highly-reflective snow can do more than give you an unfortunate tan-line; extended exposure can cause photokeratitis—snow blindness—with symptoms ranging from discomfort to temporary loss of vision. Intuitively we would expect diurnal Arctic mammals to have eyes that filter—not transmit—UV light. Overexposure to UV light could explain why cataracts are common among domesticated reindeer (Plummer & Ledbetter, 2022), but not the absence of photokeratitis, which hints at photoprotective mechanisms in the eye, such as the upregulation of ascorbic acid (Ringvold et al., 2003). The mystery deepens.

Recently, researchers have focused on the violet-blue color of twilight––known as the “blue hour” among photographers. Rich cobalt skies are created when setting sunlight travels through a thicker band of atmosphere, which selectively attenuates greenish wavelengths (Figure 1). A fleeting phenomenon in many places, twilight fills 8–11 h of each day from September to April for animals living above 70° latitude (Fosbury & Jeffery, 2022). Twilight-tapetum color matching predicts enhanced vision under dim light, including UV color discrimination (Figure 1), and it is tempting to link the evolution of this trait to the detection of UV-absorbing wolves against a background of UV-reflecting snow (Fosbury & Jeffery, 2022; Hogg et al., 2011). White wolves are well camouflaged on white landscapes; unless the prey are sensitive to UV light. It is an appealing hypothesis, but wolves in circum-Arctic habitats subsist on a variety of ungulate species––moose (*Alces alces*), muskoxen (*Ovibos moschatus*), roe deer (*Capreolus capreolus*)––which predicts similar selective pressures on their visual systems (Dominy & Harris, 2022). Studying the eyes of these animals may prove rewarding but, to date, there is little indication they share reindeers’ distinctive visual abilities.

In contrast to every other species of ruminant, reindeer graze on lichens, especially during winter. The idea that reindeer use UV vision to detect vegetation amid snow was suggested almost a decade ago, with evidence that vascular plants––but not lichens––are visually distinctive in snow (Tyler et al., 2014). However, the authors investigated *Ophioparma*, a flat, dry, rock-growing lichen that is not eaten by reindeer. Highly diverse in color and form, lichens vary in their propensity to reflect UV light (Figure 2), a fact that impelled us to study *Cladonia rangiferina*, a nomen that celebrates its ecological association with reindeer (*Rangifer tarandus*).

**Figure 2.**
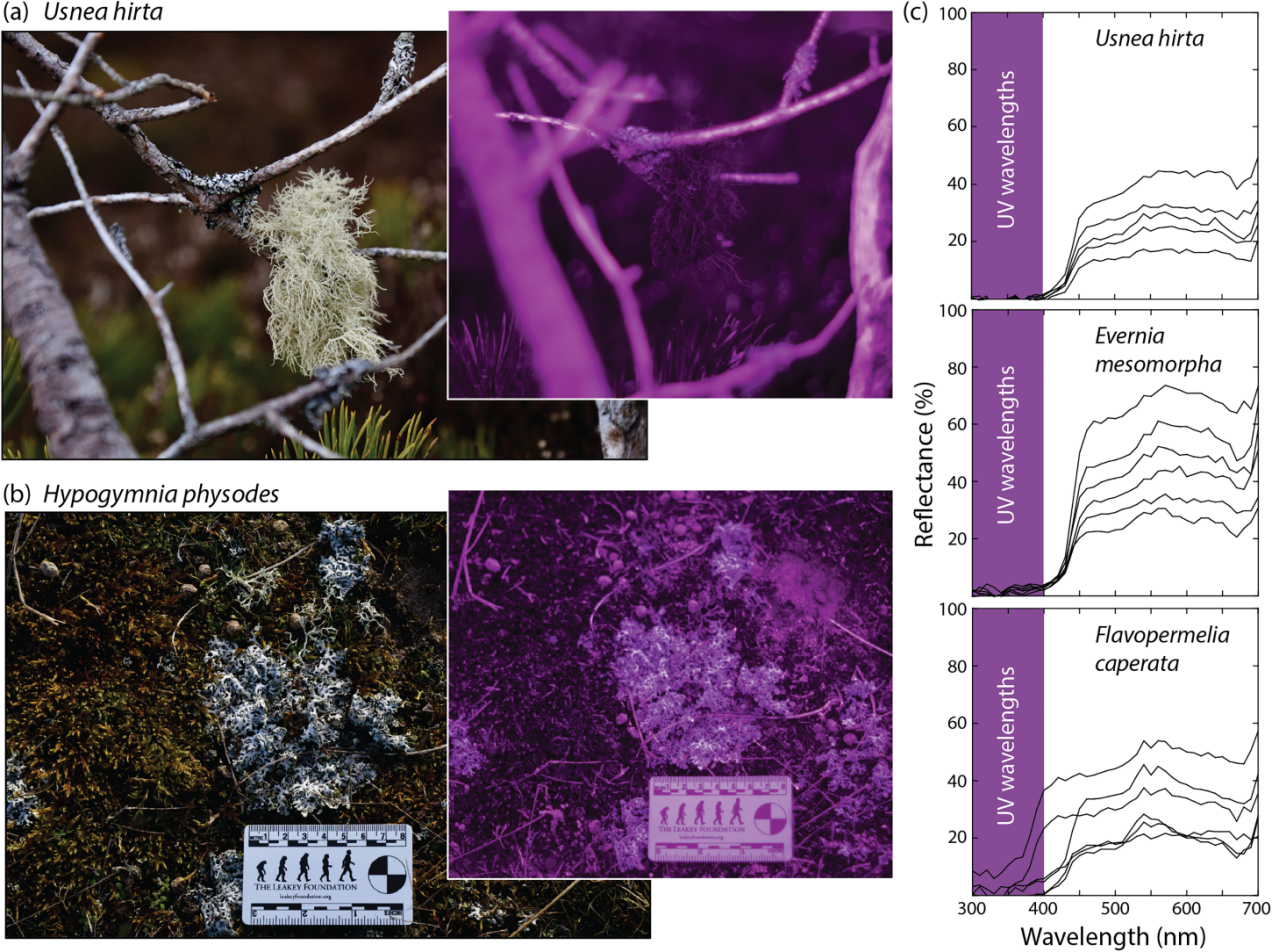
Strong UV absorbance is exemplified by (a) *Usnea hirta* (bristly beard lichen). Other species are moderately UV-reflective, such as this squamule mat of (b) *Hypogymnia physodes* (monk's hood lichen). Photographs were obtained with a full-spectrum converted Canon EOS RP camera outfitted with a UV compatible lens (Yungnuo EF 50 mm f/1.8) and UV bandpass filter (Kolari Vision, Raritan, NJ). (c) Variable levels of UV absorbance are evident in the reflectance spectra of *U. hirta*, *Evernia mesomorpha* (boreal oakmoss lichen), and *Flavoparmelia caperata* (common greenshield). Data were collected with a Jaz spectrometer and expressed relative to a WS-1 reflectance standard (Ocean Optics, Dunedin, FL).

*Cladonia* is a shrubby ground-dwelling taxon that forms deep beds. In winter, reindeer expose beds by digging (“cratering”), a process that accelerates snow melt and visibility on winter landscapes (Figure 1a). For us, pale beds of *Cladonia* were sometimes difficult to discern from patches of spring snowmelt, but our spectral data highlight the value of color discrimination between 330 and 370 nm (Figure 3), UV wavelengths that correspond to a secondary peak in twilight irradiance (Figure 1b). Taken together, our limited study of lichens suggests chromatic conspicuousness to reindeer eyes under twilight conditions. They also cast new light on the benefits of a luminescent nose––it may light the way for Santa to see by, but it is Rudolph's blue-eyes that allow him to find dinner after a long Christmas season.

**Figure 3.**
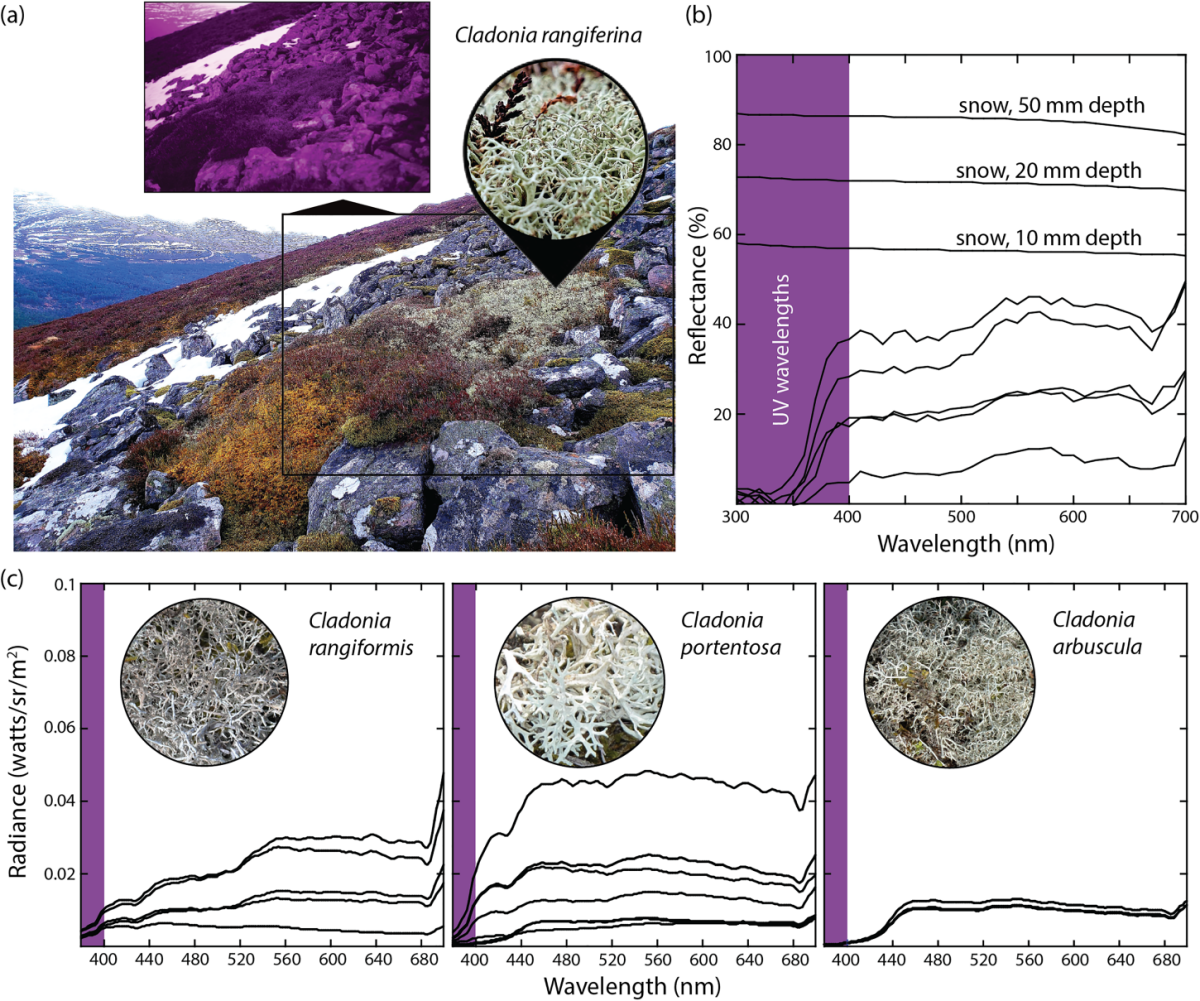
(a) Bed of *Cladonia rangiferina* (reindeer moss) in Cairngorms National Park, Scotland. Its UV absorbance relative to adjacent snow is evident in the UV photograph (inset). (b) The reflectance spectra of snow (data source: Warren, 2019) and *C. rangiferina* (see Figure 2 for methods) raise the possibility of color discrimination between 330 and 370 nm, UV wavelengths that correspond to a secondary peak in twilight irradiance (Figure 1b). (c) Radiance spectra from additional species of *Cladonia* were obtained in Tentsmuir National Nature Reserve, Scotland (taxonomy follows Dobson, 2018). Spectra were collected with a PR-670 telespectroradiometer (Photo Research, Syracuse, NY) under overcast natural light at 45° to each surface.

Linking UV visual sensitivity to feeding ecology raises tantalizing questions of how *Cladonia* might simultaneously protect reindeer eyes from UV damage. *Cladonia* has impressive antioxidant properties (Kosanić et al., 2014), and its combination with other favored foods––namely, the buds and leaves of Arctic willow (*Salix arctica*) and dwarf birch (*Betula nana*)––both of which have exceedingly high levels of Vitamin C (Rodahl, 1944), could provide a diversified suite of protective measures. Whatever the case, our essay has far-reaching practical applications by suggesting that orange juice and carrots are ideal treats for supplementing reindeer on Christmas Eve.
